# Editorial: Entrainment and responses to rhythmic stimulation during development

**DOI:** 10.3389/fpsyg.2023.1189054

**Published:** 2023-04-28

**Authors:** Stefanie Peykarjou, Stefanie Hoehl, Arnaud Leleu, Aliette Lochy, Viola Macchi Cassia

**Affiliations:** ^1^Department of Psychology, Heidelberg University, Heidelberg, Germany; ^2^Department of Developmental and Educational Psychology, Faculty of Psychology, University of Vienna, Vienna, Austria; ^3^Development of Olfactory Communication and Cognition Laboratory, Centre des Sciences du Goût et de l'Alimentation, Université de Bourgogne, CNRS, INRAe, Institut Agro, Dijon, France; ^4^Institute of Cognitive Science and Assessment (COSA), Department of Behavioral and Cognitive Sciences (DBCS), Faculty of Humanities, Social and Educational Sciences (FHSE), University of Luxembourg, Esch-sur-Alzette, Luxembourg; ^5^Department of Psychology, University of Milano-Bicocca, Milan, Italy; ^6^NeuroMI, Milan Center for Neuroscience, Milan, Italy

**Keywords:** FPVS, frequency tagging, oscillations, SSVEPs, synchronization, development

Rhythms are omnipresent in physiological processes, sensory inputs and social interactions. Humans respond to external rhythms with synchronization, which can be observed across multiple levels (e.g., behavior, brain, and physiology). Given their ubiquitous nature, utilizing rhythmic stimulation to characterize development is beneficial in many domains, including cognition, perception, and motor functioning.

This Research Topic brings together current research on behavioral and brain responses to rhythmic stimulation in development across these domains. This ultimately helps to broaden our understanding of neural entrainment (e.g., Bánki et al.), of the links between rhythms and cognitive or behavioral development (e.g., Frischen et al.; Yu et al.), and of the methods employing rhythmic stimulation to investigate early development (e.g., Peykarjou). Since rhythmic responses can be conceived as entrainment of endogenous oscillators to external rhythms (e.g., Notbohm et al., 2016), or periodic evoked responses (Capilla et al., 2011), “entrainment” is broadly defined here as responses elicited by a corresponding external driving rhythm.

Several contributions in this Research Topic focus on theoretical advancements regarding relevant concepts and relations of rhythms with development (Bánki et al.; Bowsher-Murray et al.; Frischen et al.). Bánki et al. provide a discussion of neural entrainment in contrast to stimulus tracking and argue that evidence in favor of entrainment is accumulating. However, this evidence largely comes from research with adults, and establishing the presence of neural entrainment in early development requires adapting current methods. The authors call for studies aligning the stimulation frequency to individual internal oscillations of participants and investigating how external rhythms modulate behavioral performance.

The relation between rhythm processing, cognitive and motor abilities is analyzed by Frischen et al. who discuss predictive processing (Friston, 2005) as the mechanism that might link these functions. Studies showing substantial correlations between rhythm processing and language ability, motor skills and executive functions are summarized. Predictive processing in beat perception, anticipating temporally ordered sound events, is suggested to stimulate prediction in other domains, providing a link explaining commonalities in development.

Lau et al. provide support for the view that early links between language (i.e. vocalizations) and cognition may be subserved by infants' perceptual sensitivity to rhythmic elements. Drawing on evidence from four-month-olds showing that language may facilitate categorization (Ferry et al., 2013), the authors employ supervised machine learning to show that rhythm-related features of acoustic stimuli and temporal envelope components may be vital for supporting cognition.

In their contribution, Bowsher-Murray et al. approach the role of rhythm in development by focusing on interpersonal synchrony, i.e., the “tendency for social partners to temporally coordinate their behavior when interacting”. They provide an overview of components underpinning successful non-verbal interpersonal synchrony, including social orienting, action prediction and motor behavior, monitoring and error detection, pointing out that more work on the potentially bidirectional nature of behavioral and neural entrainment is required. Indeed, spanning these levels of analysis was accomplished across contributions in this Research Topic but should be tackled within studies in the future.

Behavioral entrainment is addressed in three studies investigating the development of rattle-shaking and drumming (Laudanska et al.; Rocha and Addyman; Yu et al.). The ability to coordinate drumming movements to match external rhythms was investigated in toddlers between 18 and 30 months of age (Rocha and Addyman; Yu et al.). The beginnings of rhythmic behavior are explored by Laudanska et al., who investigate coupling between infants' arm movements longitudinally from four to twelve months. They document infants' motivation for rhythmic behavior from early on, as participants similarly attempt rattle-shaking across all ages. With age, left and right arm movements become more coupled during rattle-shaking independent of age, demonstrating how synchronization diffuses throughout the body. Yu et al. demonstrate that by 24 months, children begin to slow down their spontaneous motor tempo toward the rhythm provided by a drumming partner, but successful synchronization is only achieved by 30 months. Rocha and Addyman employ a novel tool, deep learning-based OpenPose, to automatically estimate drumming behavior from home-recorded videos. Both studies show that toddlers between 24 and 30 months of age are strongly motivated to engage in synchronous movement, as they adapt their drumming tempo away from their intrinsic rate of movement to match either a social interaction partner, a robot (Yu et al.), or the video of a drumming hand (Rocha and Addyman).

The research by Frey et al. applies rhythmic stimulation as a tool for facilitating development. In a training study with preschool children, they demonstrate correlations between children‘s rhythmic and literacy skills and between rhythm synchronization and pen pressure. Training provided in their study effectively improves rhythmic abilities, however these improvements do not transfer to literacy or graphomotor development. Further research is needed to evaluate whether rhythmic training may causally support development in other domains.

Other studies assembled in this Research Topic employ responses to rhythmic stimulation coupled with neural measures to examine auditory and visual processing (Attaheri et al.; Bertels et al.; Cantiani et al.; Pescuma et al.; Peykarjou; Poncet et al.). In these studies, rhythmic stimulation is mainly employed as a tool for exploring perceptual and cognitive development without necessitating a behavioral response.

Auditory processing is investigated by Cantiani et al. who employ frequency tagging with complex music and speech stimuli. The authors report similar neural entrainment to auditory stimuli, particularly music, in eight-month-old infants and adults. They infer that neural synchronization and tempo flexibility develop before synchronization abilities become apparent in behavior. Attaheri et al. also compare neural synchronization to speech stimuli between adults and infants (four to eleven months) and find commonalities across development. Participants track sung speech in delta and theta bands, but specific peak-frequencies differ between the two populations. Together, these studies suggest that infant and adult brains utilize similar cortical mechanisms to track linguistic inputs, but the interplay between these mechanisms varies with age and language experience.

Four studies employ the Fast Periodic Visual Stimulation (FPVS) oddball paradigm (Liu-Shuang et al., 2014) with visual stimuli. Infants' developing ability to discriminate facial expressions from 3.5 to 7 months is investigated by Poncet et al.. Significant expression discrimination is observed from 3.5 months, with partly diverging topographies across facial expressions and age. Responses for emotional faces among neutral ones are recorded at right occipito-temporal sites, similar to adults (Poncet et al., 2019), suggesting that the discrimination of neutral vs. expressive faces is subserved by similar networks across development.

Bertels et al. investigate characteristics of a potentially inborn mechanism to detect snakes, a species posing an evolutionarily important threat to humans. EEG responses in six-to-eleven-month-olds reveal that they detect snakes among other animals, and this effect is not influenced by color. However, responses increase with infant age, which is interpreted as a specific visual development for evolutionary-relevant shapes in complex backgrounds.

Peykarjou presents a systematic literature review on frequency tagging EEG employing the FPVS oddball paradigm and examines the effects of analysis decisions in a large sample of infants. Based on the assembled evidence, recommendations regarding duration, number of conditions, sequence- and participant-retention are made. Overall, evidence indicates that analysis decisions should be tailored to the age-group.

Pescuma et al. use frequency tagging to investigate a developing cultural skill, morpheme identification. They adapt an established paradigm (Lochy et al., 2016a,b) to MEG and demonstrate morpheme identification in developing readers that is overall similar to adults. Neural responses are primarily present for word stems in children and word suffixes in adults, suggesting an influence of accumulated reading experience.

This Research Topic assembles a variety of findings demonstrating the importance of rhythms for development, and their usefulness for elucidating cognitive, visual, auditory, and motor functions. Moreover, it provides readers with an overview of methodological advancements for infant studies, such as wearable motion trackers (Laudanska et al.), an online version of a measure for drumming synchrony (Rocha and Addyman), or guidelines for frequency tagging EEG categorization tasks (Peykarjou). By integrating findings across the different contributions, several conclusions can be drawn. First, rhythms provide both a window into development across different domains and a linking concept for understanding the interplay of development and environmental influences. Second, across developmental domains and levels of analysis, rhythms play an important role that we only begin to understand. Third, at both conceptual and empirical levels, further work is required to leverage the potential of rhythmic stimulation and entrainment for developing a more comprehensive picture of how behavior and brain processing develop across domains.

## Author contributions

SP wrote the initial draft. All authors listed have made a substantial, direct, and intellectual contribution to the work and approved it for publication.
